# LINC00152 induced by TGF-β promotes metastasis via HuR in lung adenocarcinoma

**DOI:** 10.1038/s41419-022-05164-2

**Published:** 2022-09-07

**Authors:** Wei Xu, Linna Chen, Jiheng Liu, Zhezhe Zhang, Ranran Wang, Qianqian Zhang, Huiting Li, Juanjuan Xiang, Li Fang, Ping Xu, Zheng Li

**Affiliations:** 1 NHC Key Laboratory of Carcinogenesis, Hunan Cancer Hospital and the Affiliated Cancer Hospital of Xiangya School of Medicine, Central South University, Changsha, Hunan China; 2 Academy of Medical Science, Zhengzhou University, Zhengzhou, Henan China; 3 Department of Hematology & Oncology, First Hospital of Changsha, Changsha, Hunan China; 4 Department of Cardiovascular Surgery, Second Xiangya Hospital, Central South University, Changsha, Hunan China; 5 Departments of Respiratory and Critical Care Medicine, Peking University Shenzhen Hospital, Shenzhen, Guangdong China

**Keywords:** Cell biology, Cancer

## Abstract

Lung adenocarcinoma (LUAD) is one of the main causes of cancer-related mortality, with a strong tendency to metastasize early. Transforming growth factor-β (TGF-β) signaling is a powerful regulator to promote metastasis of LUAD. Here, we screened long non-coding RNAs (lncRNAs) responsive to TGF-β and highly expressed in LUAD cells, and finally obtained our master molecular LINC00152. We proved that the TGF-β promoted transcription of LINC00152 through the classical TGF-β/SMAD3 signaling pathway and maintained its stability through the RNA-binding protein HuR. Moreover, LINC00152 increased ZEB1, SNAI1 and SNAI2 expression via increasing the interactions of HuR and these transcription factors, ultimately promoting epithelial-mesenchymal transition of LUAD cell and enhancing LUAD metastasis in vivo. These data provided evidence that LINC00152 induced by TGF-β promotes metastasis depending HuR in lung adenocarcinoma. Designing targeting LINC00152 and HuR inhibitors may therefore be an effective therapeutic strategy for LUAD treatment.

## Introduction

Lung cancer is one of the most common malignant tumors threatening human health, which results cancer-associated mortalities in men (21.5%) and women (13.7%) worldwide [1–3]. Lung adenocarcinoma (LUAD) accounts for approximately 40% of all lung cancer cases which incidence has rapidly increased in recent years [4]. Despite more than 1/3 of LUAD patients recovering after traditional treatments and molecular targeted therapy, the mortality rate is still high due to the tendency to metastasis early [5–7]. Therefore, screening of the genes related to LUAD metastasis and elucidating their molecular mechanism is vital in improving the prognosis of patients and reducing mortality.

Transforming growth factor-β (TGF-β) signaling pathway is a major inducer of EMT and facilitate cancer metastasis by targeting downstream various effector molecules [8, 9]. Like proteins, long non-coding RNAs (lncRNAs) can act as intermediates of TGF-β signaling and play an essential role in regulating the migration and invasion of tumor cells [10, 11]. For example, TGF-β induces lncRNA-ATB overexpression in hepatocellular carcinoma. ATB promotes the expression of ZEB1 and ZEB2 through competition with the miR-200 family to induce epithelial-mesenchymal transition (EMT) and invasion of hepatocellular carcinoma cells [12]. In lung cancer, lncRNA-SMASR is downregulated by TGF-β via SMAD2/3 [13]. TGF-β is an important cytokine that induces the EMT and promotes tumor progression by upregulating the expression of EMT core transcription factors, LINC00273 promotes TGF-β induced EMT through a miR-200a/ZEB1 feedback loop [14]. However, TGF-β regulated lncRNAs in lung adenocarcinoma are still not fully studied.

RNA-binding proteins (RBPs) play an essential role in RNA splicing, polyadenylation, sequence editing, RNA transportation, RNA stability and degradation, intracellular positioning, and translation control [15–17]. RBPs often establish concerted networks to regulate their target genes and to affect tumor progression. PCBP1 interacts with phosphorylated SMAD3 to regulate splicing of CD44 and promote tumor metastasis [18]. EIF2S2 specifically regulates the stability of LINC01600 in colorectal cancer cells [19]. HuR binds to lncRNA HGBC to maintain its stabilization, which overexpression promotes the proliferation and metastasis of gallbladder cancer cells [20].

In this study, we screened the lncRNAs regulated by TGF-β in LUAD and found LINC00152 regulated by TGF-β and highly expressed in LUAD cells and tissues. The LINC00152 is a long noncoding RNA of 852 nucleotides and is uniformly distributed in the cytoplasm and nucleus [21]. It has been reported that LINC00152 is a cancer-promoting gene in certain tumors, such as lung cancer [22, 23], colorectal cancer [24, 25], esophageal squamous cell carcinoma [26] and oral squamous cell carcinoma [27]. In colorectal Cancer, LINC00152 expression regulated by YAP1 and its overexpression promoted tumor cells proliferation via modulating Fascin actin-bundling protein 1 [28]. However, the molecular mechanisms of TGF-β regulating LINC00152 expression and LINC00152 promoting LUAD development remains unclear.

Here, we demonstrated that TGF-β regulated the transcription of LINC00152 through SMAD3. Furthermore, RNA-binding protein HuR directly banded with LINC00152 and TGF-β maintained the stability of LINC00152 by upregulating HuR. In particular, LINC00152 in turn affected HuR interaction with EMT associated core transcription factors (ZEB1, Snail and Slug) and enhanced their expression to promote the metastasis of LUAD.

## Results

### LINC00152 is a TGF-β responder lncRNA in LUAD

**Fig. 1:**
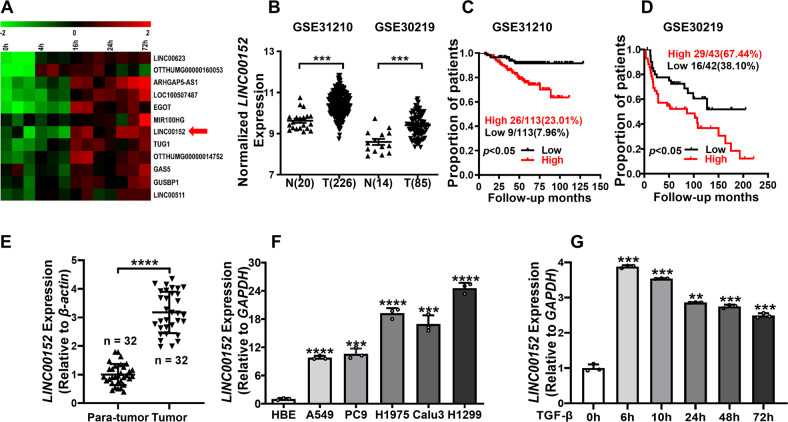
LINC00152 is overexpression in LUAD as a TGF-β induced LncRNA. **A** The heat maps of the 12 LncRNAs expression in A549 cells induced by TGF-β at different time points. **B** LINC00152 expression in human LUAD tissue samples and adjacent lung tissue samples from GSE31210 and GSE30219 data. **C**, **D** Kaplan–Meier survival analysis of LUAD dataset for overall survival from GSE31210 and GSE30219 data. Each cohort was divided into LINC00152 high (red) and low (black). **E** LINC00152 expression was analyzed by RT-qPCR in matched tumor samples compared with para-tumor samples (*n* = 32). **F** The expression of LINC00152 in LUAD cells compared to human bronchial epithelial (HBE) cells. **G** A549 cells were treated with TGF-β (5 ng/mL) at different times and the expression of LINC00152 was detected by RT-qPCR. Data have been presented as the mean ± SD of three independent experiments (**p < 0.01; ***p < 0.001; ****p < 0.0001. Student’s *t*-test).

To obtain TGF-β responded lncRNAs in LUAD, we compared the different genes expression in A549 cells treated with or without TGF-β for 72 h using GSE17708 dataset (Fold Change ≥ 2*, p* ≤ 0.05). We found 85 differentially expressed lncRNAs which included 52 upregulated and 33 down-regulated genes after TGF-β treatment. Furthermore, 12 of 52 potential TGF-β induced lncRNAs were highly expressed in lung adenocarcinoma tissues. They included ARHGAP5-AS1, LOC100507487, MIR100HG, LINC00623, EGOT, LINC00152, TUG1, OTTHUMG00000014752, GAS5, GUSBP1, OTTHUMG00000160053, and LINC00511. The expression heat map of these 12 lncRNAs at different time points after TGF-β treatment in A549 cells is shown in Fig. 1A. Further analysis of the expression level and its relationship with the prognosis of LUAD patients were conducted on these 12 lncRNAs using GSE31210 and GSE30219 (Figs. S1 and S2). The LINC00152 was highly expressed in both LUAD tissues and negatively correlated with the survival prognosis of patients in these two datasets (Fig. 1B–D).

The study detected the expression of LINC00152 in 32 pairs of LUAD tissue and paracancerous samples, and results showed that LINC00152 was overexpression in tumor tissues (Fig. 1E). Compared with human bronchial epithelial cells HBE, the LINC00152 expression was higher in A549, PC9, H1975, Calu3, and H1299 cell lines (Fig. 1F). After induction of TGF-β (5 ng/ml) in A549 cells, the expression of LINC00152 was up-regulated to 3.8-fold at 6 h, 3.5-fold at 10 h, and 2.5-fold at 72 h, respectively. The results confirmed that the expression of LINC00152 activated and maintained by TGF-β in A549 cells (Fig. 1G). Therefore, we focus on mechanisms of how TGF-β regulates LINC00152 expression and the functions of LINC00152 in LUAD progression.

### TGF-β regulates transcription of LINC00152 via SMAD3

**Fig. 2:**
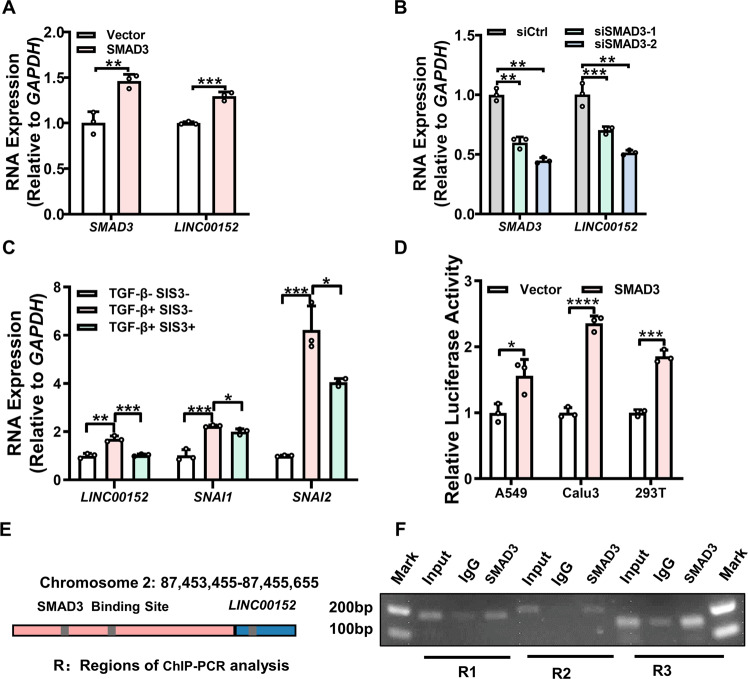
LINC00152 is a direct transcriptional target of SMAD3. **A**, **B** The expression of LINC00152 was texted by RT-qPCR in the SMAD3-silenced or overexpressed A549 cells. **C** The expression of LINC00152 was detected with the addition of TGF-β with or without SMAD3 inhibitor SIS3. **D** Dual-luciferase reporter assays detected the activity of the LINC00152 promoter in the SMAD3-overexpressing A549, Calu3 and 293 T cells. Luciferase activity was normalized to Renilla. **E** Schematic illustrations showing the predicted locations of three SMAD3-binding sites in LINC00152 promoter. **F** ChIP assay using the specific SMAD3 antibodies, then LINC00152 associated with three promoter regions was detected by PCR using specific primers. Data have been presented as the mean ± SD of three independent experiments (**p < 0.01; ***p < 0.001; ****p < 0.0001. Student’s *t*-test).

Using the online software meme-FIMO, we predicted three major SMAD3 binding sites on the LINC00152 promoter, located at −949/−940 (site 1), −734/−725 (site 2), and +103~+112 (site 3) nucleotides, respectively (Table S1). We observed a significant upregulation of the LINC00152 expression after SMAD3 overexpression and SMAD3 knockdown decreased the expression of LINC00152 in A549 cells (Fig. 2A, B). Furthermore, A549 cells were treated with p-SMAD3 small molecule inhibitor SIS3 combined with or without TGF-β. The results showed the expression of LINC00152 induced by TGF-β was inhibited by SIS3 which was similar to SNAI1 and SNAI2 change trend (Fig. 2C).

To confirm that LINC00152 was a direct transcriptional target of SMAD3, the promoter region of LINC00152 (−2000 to +200) was cloned into the pGL3-enhancer luciferase reporter vector. In A549, Calu3, and HEK293T cells, overexpression of SMAD3 markedly increased the LINC00152 promoter activity but did not enhance the activity of vector control (Fig. 2D). ChIP-PCR assay revealed the existence of SMAD3 specific binding region located on the LINC00152 promoter at position −734 to −725 relative to the start of transcription (Fig. 2E, F). These findings showed that TGF-β/SMAD3 directly regulates LINC00152 transcription in LUAD.

### TGF-β regulates stability of LINC00152 via HuR

**Fig. 3:**
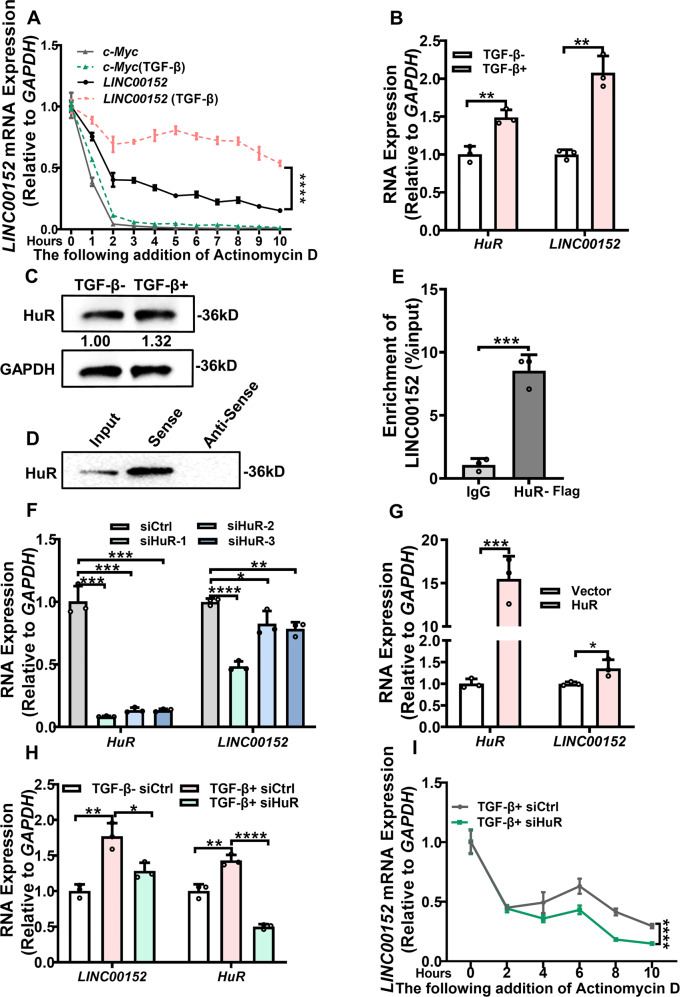
TGF-β regulates mRNA stability of LINC00152 via HuR. **A** Effect of TGF-β on LINC00152 stability detected by RT-qPCR. Actinomycin D was used to inhibit transcription and c-Myc was used as control. Results were normalized to time zero for each construct. **B**, **C** The expression of HuR and LINC00152 were tested under TGF-β stimulation. **D** RNA pull-down analysis was performed with biotin-labelled sense strand and anti-sense strand of LINC00152. Immunoprecipitated fractions were analyzed by western blotting using HuR. **E** A549 cell lines were transfected with HuR-Flag plasmids. The RIP assay was performed after thirty-six hours. The relative fold of LINC00152 binding with HuR was detected with RT-qPCR. **F**, **G** After transfection with siHuR or HuR overexpression plasmids in A549 cells, RT-qPCR was used to detect the expression level of LINC00152 mRNA. **H** RT-qPCR was used to detect the expression of HuR and LINC00152 after treatment with TGF-β and siHuR. **I** LINC00152 stability was detected by RT-qPCR at different time point after treatment with TGF-β and siHuR. Actinomycin D was used to inhibit transcription. Data have been presented as the mean ± SD of three independent experiments (*p < 0.05; **p < 0.01; ***p < 0.001; ****p < 0.0001. Student’s *t*-test).

The overexpression of lncRNA maybe result from transcription enhancing or increased mRNA stability [29]. We observed LINC00152 maintained high expression in A549 cells to 72 h of TGF-β treatment in above experiment. Therefore, the A549 cells were added with actinomycin D and LINC00152 stability was detected after TGF-β stimulation. The results showed the half-life of LINC00152 obviously increased after TGF-β treatment compared with C-MYC (Fig. 3A). We hypothesized that some RNA-binding proteins maybe participate in this regulation process.

Using PAR-CLIP datasets in POSTAR2, we predicted some potential interaction between a series of RNA-binding proteins (RBPs) and LINC00152 (Fig. S3A). Using GEPIA website, we noticed an overexpression of HuR in LUAD tumors that was associated with poor prognosis (Fig. S3B, C). Intriguingly, HuR has been reported to be induced by TGF-β in prostatic cancer and to increase the stability of VEGF mRNA [30]. We tested that the expression of HuR and LINC00152 both increased after TGF-β stimulation (Fig. 3B, C). The result showed that HuR was pulled down by the biotin-labeled LINC00152, but not antisense probe (Fig. 3D). RIP experiment showed that LINC00152 was enriched in the anti-HuR group compared with the IgG control group (Fig. 3E). These findings reveal a direct binding relationship between HuR and LINC00152.

Moreover, we found that knocking down HuR reduced the mRNA level of LINC00152 in A549 cells (Fig. 3F). Similarly, the expression of LINC00152 increased after HuR overexpression (Fig. 3G). In turn, LINC00152 had no influence on HuR expression in mRNA and protein level (Fig. S3D, E). Notably, the increase in the expression of LINC00152 induced by TGF-β was down-regulated after HuR inhibition (Fig. 3H). Meanwhile, the stability of LINC00152 decreased at this condition (Fig. 3I). These results suggest that TGF-β maintains the stability of LINC00152 through HuR and thus keeps its expression level high in LUAD cells.

### LINC00152 promotes invasion and EMT of LUAD cells through HuR

**Fig. 4:**
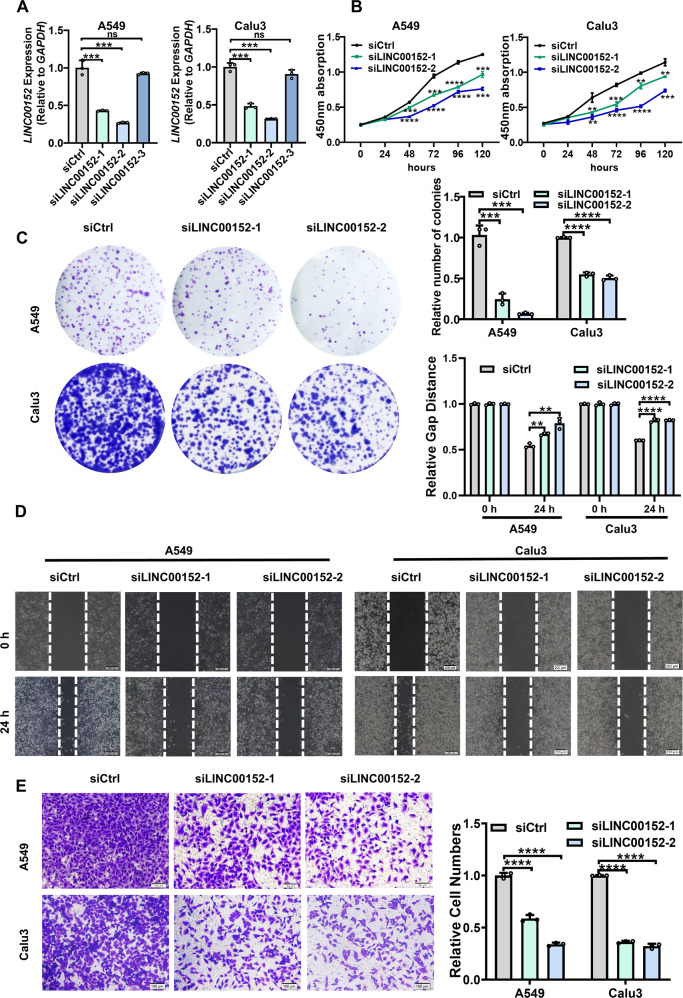
Knockdown of LINC00152 inhibits the survival, invasion and migration of LUAD cells in vitro. **A** LINC00152 expression was detected after transfection of three small interfering RNAs (siRNA) into A549 and Calu3 cells. **B** Cell Counting Kit-8 (CCK-8) assays were performed in A549 and Calu3 cells silenced for LINC00152. **C** Silencing of LINC00152 inhibits the colony formation capability of A549 and Calu3 cells. **D** The migratory potential of LINC00152 knockdown together with their controls was analyzed in a wound-healing assay. Scale bar: 200 μm. **E** The invasive potential of silenced LINC00152 together with their controls was analyzed by transwell assay. Scale bar: 100 μm. Data shown are representative images or expressed as the mean ± SD of each group from three separate experiment (**p < 0.01; ***p < 0.001; ****p < 0.0001. Student’s *t*-test).

**Fig. 5:**
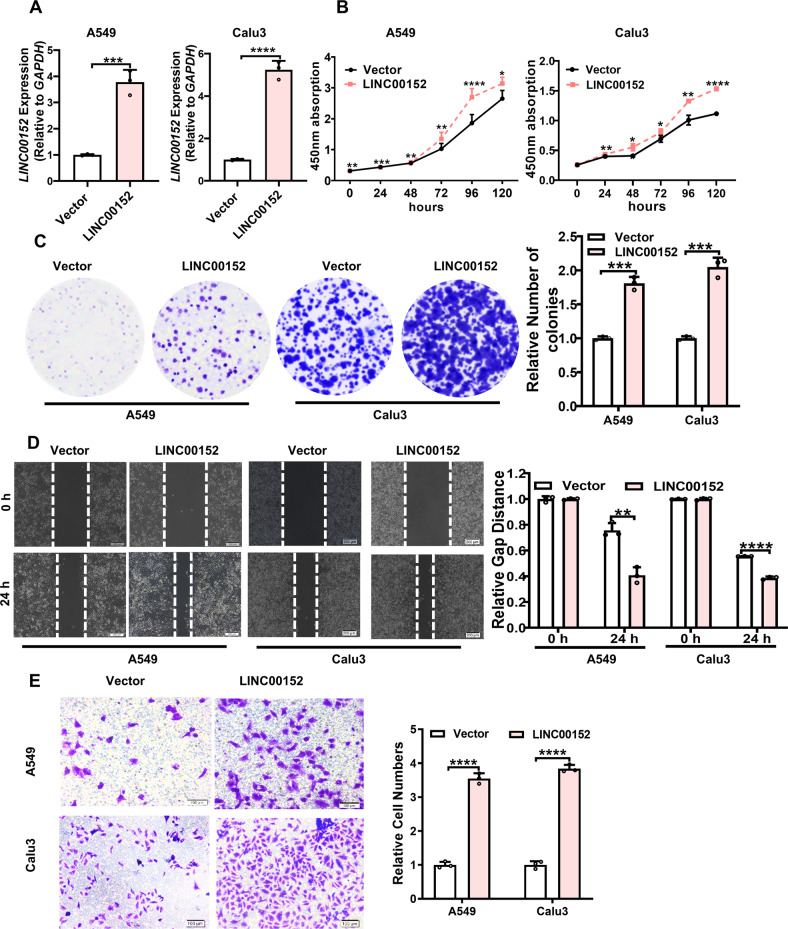
Overexpression of LINC00152 promotes the survival, invasion and migration of LUAD cells in vitro. **A** LINC00152 expression was detected after transfection of pcDNA3.1-LINC00152 plasmid into A549 and Calu3 cells. **B** Cell Counting Kit-8 (CCK-8) assays were performed in A549 and Calu3 cells with overexpressing LINC00152. **C** Overexpression of LINC00152 promotes the colony formation of cells. **D** The migratory potential of LINC00152 overexpression together with their controls was analyzed in a wound-healing assay. Scale bar: 200 μM. **E** The invasive potential of LINC00152 overexpression together with their controls was analyzed by a transwell assay. Scale bar: 100 μm. Data shown are representative images or expressed as the mean ± SD of each group from three separate experiment (**p < 0.01; ***p < 0.001; ****p < 0.0001. Student’s *t*-test).

To assess the biological function of LINC00152 in cells, siRNA was designed to specifically inhibit LINC00152 gene expression (Fig. 4A). CCK8 experiments and Colony formation assays showed that knockdown of LINC00152 inhibited the survival and colony formation capability of LUAD cells (Fig. 4B, C). Transwell experiments and scratch assays displayed that the reduction of LINC00152 inhibited invasion and migration of transfected cells (Fig. 4D, E). On the other hand, we constructed the overexpression plasmid pcDNA3.1-LINC00152 with the empty vector as a control (Fig. 5A). CCK8 and Colony formation results revealed an enhancement of the survival and colony formation capacity of LUAD cells by the overexpression of LINC00152. Transwell experiments and scratch experiments portrayed that overexpression of LINC00152 promoted the invasion and migration ability of A549 and Calu3 cells (Fig. 5B–E).

**Fig. 6:**
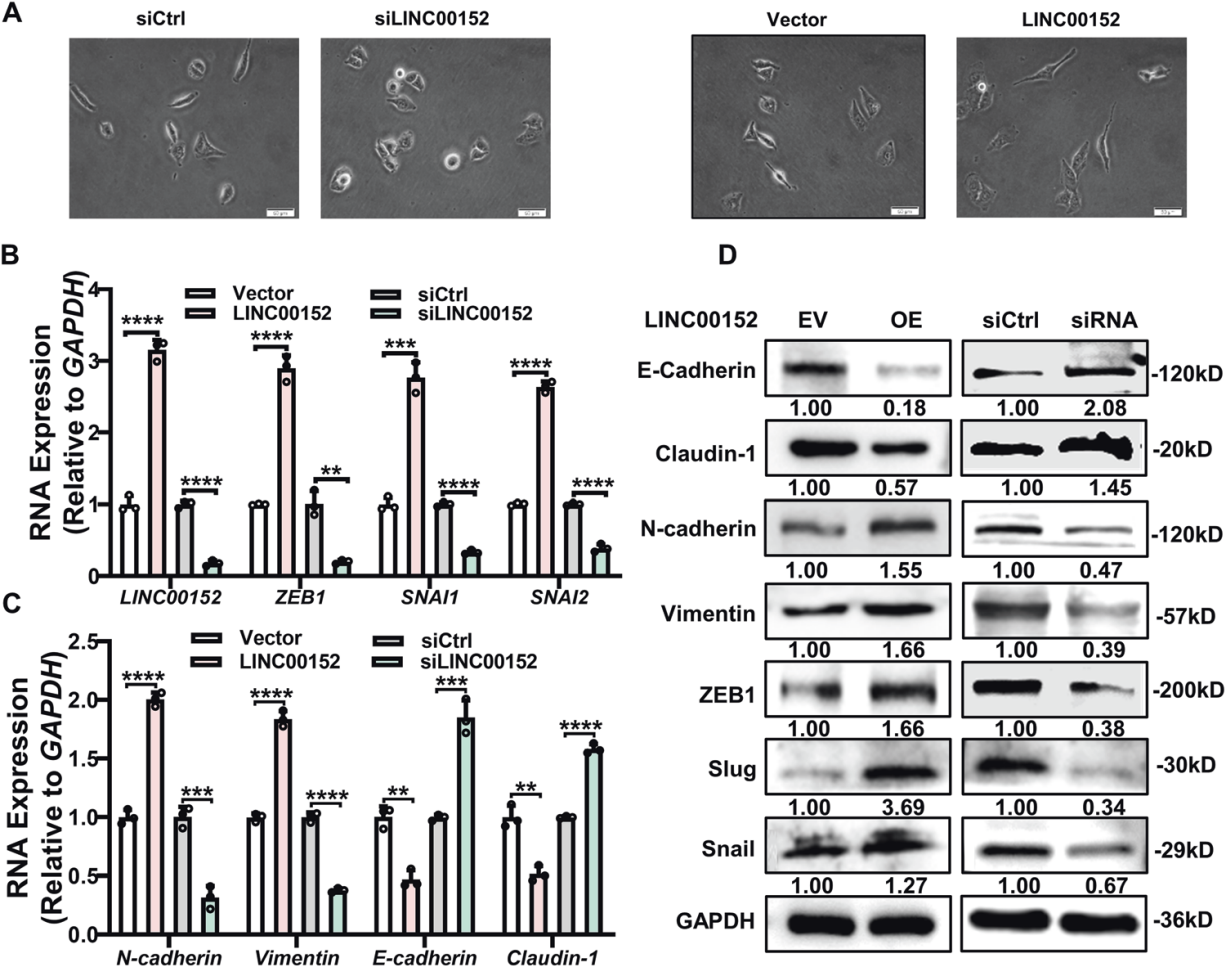
LINC00152 promotes EMT of LUAD cells. **A** Phase-contrast microscopy images of A549 cells knockdown or overexpressing LINC00152. **B**–**D** RT-qPCR and Western blot analysis of the EMT associated genes (E-cadherin, N-cadherin, Vimentin, Claudin-1, SNAI1, SNAI2 and ZEB1) expression under which LINC00152 was silenced in Calu3 cells or overexpressed in A549 cells. Data have been presented as the mean ± SD of three independent experiments (**p < 0.01; ***p < 0.001; ****p < 0.0001. Student’s *t*-test).

After LINC00152 inhibition, we observed rounding of A549 cells and disappearance of pseudopodia. However, after the expression of LINC00152, the pseudopodia of the cells became longer and the intercellular adhesion was weakened. (Fig. 6A). Furthermore, we detected the expression of EMT-related transcription factors and markers after LINC00152 expressional altered. It was found that after LINC00152 overexpression in A549 cells, both the transcription factor ZEB1, SNAI1, SNAI2 and the mesenchymal markers N-cadherin and Vimentin were upregulated, while the epithelial markers E-cadherin and Claudin-1 were downregulated. In contrast, LINC00152 knocking down induced opposed expression changes both at the protein and RNA level in Calu3 cells (Fig. 6B–D). The above experiments suggested that LINC00152 promotes EMT in lung adenocarcinoma cells.

**Fig. 7:**
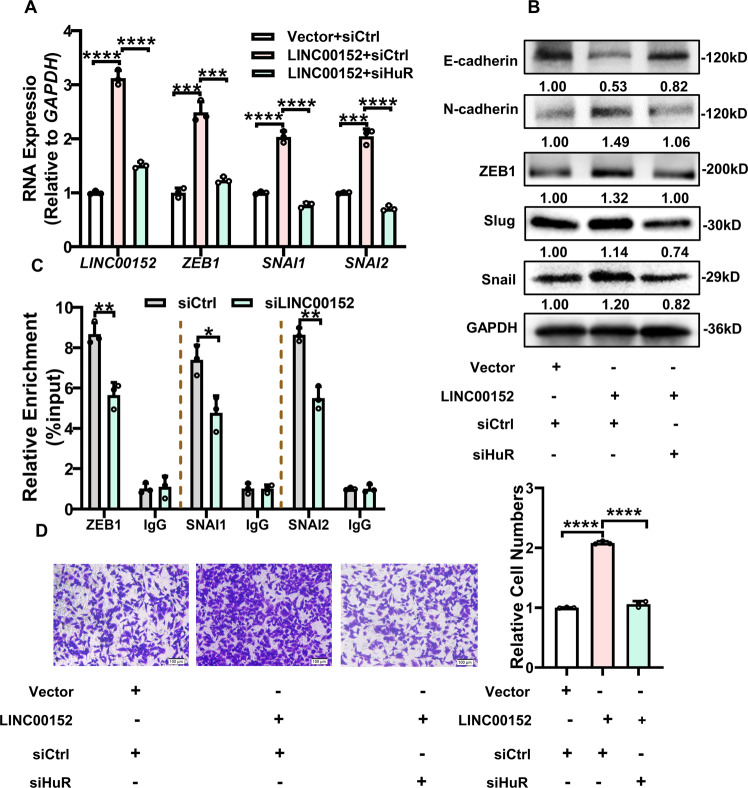
LINC00152 promotes EMT and invasion depending HuR. **A**, **B** RT-qPCR and western blot was used to detect the expression of EMT associated genes (ZEB1, SNAI1, SNAI2, E-cadherin, N-cadherin) after overexpression LINC00152 and knockdown of HuR. **C** RIP assay of HuR binding RNAs of EMT related genes with an anti-Flag antibody in silenced LINC00152 or control cells. Data for each individual mRNA was normalized to the IgG pull-down product of that mRNA. **D** The invasive ability of overexpression LINC00152 and knockdown of HuR was analyzed by a transwell assay. Data have been presented as the mean ± SD of three independent experiments (**p < 0.01; ***p < 0.001; ****p < 0.0001. Student’s *t*-test).

In pancreatic cancer, HuR can bind to the SNAI1 mRNA and upregulation its expression to promote metastasis of tumor [31]. We speculated that LINC00152 regulation EMT ability maybe through interaction with HuR. After transfection of LINC00152 plasmid with siHuR in A549 cells, we found that HuR knocking down could decrease the high expression of ZEB1, SNAI1 and SNAI2 caused by LINC00152 overexpression. In this processing, N-cadherin expression showed same variation trend while E-cadherin expression exhibited opposite results (Fig. 7A, B). A549 cells were transfected with HuR-Flag and RIP assay was performed using Flag antibodies. We observed that HuR could bind to the mRNA of ZEB1, SNAI1 and SNAI2. Importantly, LINC00152 downregulation decreased the binding of HuR with ZEB1, SNAI1 and SNAI2 (Fig. 7C). We also observed that A549 cells invasive ability enhanced by LINC00152 overexpression was reversed by HuR knocking down (Fig. 7D). These results demonstrated that LINC00152 accelerate EMT processing via regulation interactions between HuR and EMT core transfactors.

### LINC00152 inhibits LUAD growth and metastasis in vivo

**Fig. 8:**
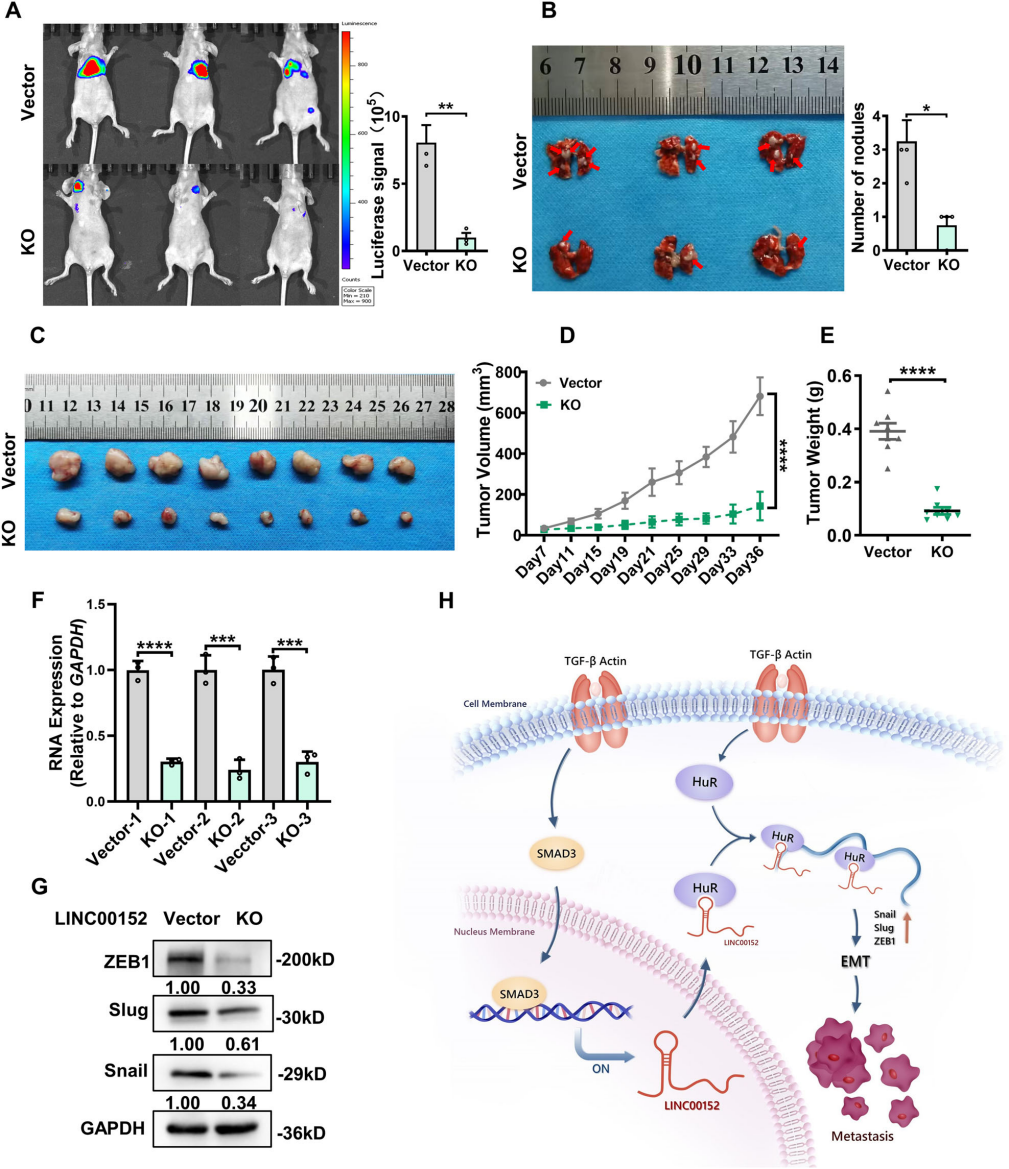
Knocking out of LINC00152 by CRISPR/Cas9 system inhibits tumor growth and metastasis in vivo. **A** Vector control or LINC00152 KO cell were intravenously injected into nude mice. Luciferase signal intensities of each group were examined at 8 weeks, (*n* = 3 per group). **B** Images of visible nodules on the lung surface. The number of lung metastatic nodules on each lung surface was quantified. **C** Images of subcutaneous tumor formation in nude mice at 5 weeks. Mice were injected with 5 × 10^6^ Vector control or LINC00152 KO A549 cell. **D**, **E** Tumor volumes and tumor weights were measured for each group (*n* = 8 per group). **F** RT-qPCR for LINC00152 expression in xenograft tumor tissues of Vector control or LINC00152 KO group. **G** Western blot for EMT associated genes (ZEB1, Snail, Slug) in xenograft tumor tissues of Vector control or LINC00152 KO group. **H** Schematic diagram of the molecular mechanism of regulation of LINC00152 expression and its function in LUAD. All the data have been presented as the mean ± SD of three independent experiments (*p < 0.05; **p < 0.01; ****p < 0.0001. Student’s *t*-test).

To detect LINC00152 function in vivo, we designed a pair of small guide RNAs (sgRNAs) which located outside of LINC00152 exon 1 (E1) (Fig. S4A–C). We chose #2 clone in which expression of LINC00152 was down-regulated almost 90% (Fig. S4D). The transwell experiment showed that LINC00152 knockout effectively inhibited the invasion ability of A549 cells (Fig. S4E). We found that the ZO-1 was upregulated while the ZEB1 and Slug were downregulated at the LINC00152 KO cell (Fig. S4F). In order to monitor tumor metastasis in vivo, vector control and LINC00152 KO cells were labeled with luciferase-EGFP. Then, we evaluated LINC00152 affection in metastasis using the tail vein injection model. Eight weeks after injection, we found that nude mice in LINC00152 KO group produced less lung metastases nodules compared with control A549 cells (Fig. 8A, B). We also observed that LINC00152 knockout obviously inhibited the tumor growth in vivo (Fig. 8C–E and Fig. S4G). Importantly, tumors isolated from LINC00152 KO group expressed lower levels of ZEB1, Snail and Slug than those tumors from control group in xenograft models (Fig. 8F, G). Taken together, our results support that LINC00152 is a key player in LUAD metastasis through regulation EMT core transfactors depending HuR.

## Discussion

In this study, we screened 12 lncRNAs potentially regulated by the TGF-β signaling pathway in lung adenocarcinoma tissues. Among them, TUG1 has been reported to promote proliferation and migration of pancreatic cancer cells through the regulation of EMT [32]. The GAS5 modulates smooth muscle cell differentiation via disturbing SMAD3 binding to target differentiation genes [33]. Knocking down LINC00511 can inhibit TGF-β induced migration and invasion of lung cancer cells by reducing matrix metallopeptidases expression [34]. These studies suggest that lncRNAs as important roles participate in TGF-β signaling pathway to mediate various pathophysiological processes. We focused our studies on LINC00152, which overexpression had a strong correlation with the poor prognosis of LUAD patients. LINC00152 was highly expressed in LUAD cells and LUAD tumor samples. Furthermore, we verified that TGF-β induced high expression of LINC00152 and maintain its overexpression in LUAD cells.

TGF-β mainly acts on SMAD proteins through serine/threonine kinase transmembrane receptors and activates a series of transduction signal proteins to elicit biological effects [35, 36]. In silico analysis suggests that there are three SBEs in LIN00152 promoter region. The luciferase and ChIP experiments confirmed that TGF-β/SMAD3 indeed promotes the transcription of LINC00152 in LUAD. Gene expression is not just regulated at transcription, but regulated at post-transcription and epigenetic levels [37]. Our data indicated that the stability of LINC00152 was also enhanced after the stimulation with TGF-β. It has been reported that RNA-binding proteins play an essential role in maintaining the stability of gene mRNA [38]. We predicted a series of potential LINC00152 RNA binding proteins and focused on HuR. TCGA data analysis shown that HuR was high expression in LUAD tissue and associated with poor prognosis of patients. In cardiac fibrosis, TGF-β prolonged the half-life of Secreted Frizzled Related Protein 2 (SFRP2) by enhancing the lncRNA Safe-HuR-Sfrp2 complex formation to promote fibrosis [39]. We found that TGF-β enhanced the expression level of LINC00152 and HuR simultaneously. Using RNA pull-down experiment and RIP experiment, we verified that LINC00152 directly bound with HuR. Rescue experiment revealed that knocking down HuR reversed the upregulation and stability of LINC00152 induced by TGF-β. Therefore, TGF-β regulates the transcription of LINC00152 through SMAD3, and maintains the stability of LINC00152 mRNA through HuR, hence achieving high expression of LINC00152 in lung adenocarcinoma tissues.

Our previous study showed that LncRNA CAR10 and HCP5 acted as endogenous RNA to promote LUAD cells migration and invasion by completely binding to miR-30/203 with SNAI [11, 40]. A previous article stated that LINC00152 promoted LUAD cells proliferation via interaction with EZH2 to repress IL-24 transcription [41]. In this study, we found that LINC00152 induced the EMT accompanied by three core transcription factors SNAI1, SNAI2 and ZEB1 upregulation. Furthermore, RIP experiment showed that HuR could bind with ZEB1, SNAI1 and SNAI2 mRNA. Decreasing of LINC00152 reduced HuR interactions with these three genes. We hypothesize that LINC00152 is able to form a complex with HuR and ZEB1, SNAI1 and SNAI2 mRNA, which inhibit these transfactors mRNA degradation. Further studies are required to fully decipher the LINC00152 interaction partners in later studies. Consequently, the invasive ability of A549 cells induced by high LINC00152 expression were reversed by HuR downregulation accompanied by corresponding expression changes. More importantly, we also detected ZEB1, snail and Slug downregulation in tumors isolated from LINC00152 KO group. Taken together, our data suggest that LINC00152 maybe maintain EMT associated transfactors stability via HuR to activate downstream genes and EMT phenotype in LUAD.

In conclusion, our results revealed that TGF-β regulates LINC00152 transcription via SMAD3 and maintains its stability through HuR, upregulating LINC00152 expression in LUAD tumor tissues. Moreover, LINC00152 regulates EMT core transcription factor expression via affecting HuR interactions with these genes to promote metastasis of LUAD. LINC00152 and HuR emerge as the promising target for LUAD clinical therapeutics and deserves further investigation.

## Materials and methods

### Bioinformatics analysis

To obtain TGF-β regulated lncRNAs expression data, we downloaded Gene Expression Omnibus (GEO) dataset GSE17708 [42]. This microarray (U133 Plus 2.0Affymetrix) data includes gene expression of human A549 lung adenocarcinoma cell line with TGF-β (5 ng/ml) for different time points. Using GCBI website [43], we reanalyzed the lncRNAs differentially expressed between control cells and A549 stimulated 72 h with TGF-β (fold change >2, *P* < 0.05). Then we analyzed the expression of the TGF-β upregulated lncRNAs in LUAD tissues using the bioinformatics tool “lncRNAtor”. The Kaplan–Meier survival analysis of high-expressed lncRNAs in LUAD tissue was conducted in GEO dataset GSE31210 [44] and GSE30219. GSE31210 included 226 cases of primary LUAD tissues of pathological stage I–II and 20 normal tissues. GSE30219 contains gene expression data of 293 lung tumor specimens (85 lung adenocarcinoma specimens) and 14 non-tumor lung specimens. The online analysis website JASPAR [45] and meme-FIMO [46] were used to predicate the combination between SMAD3 and LINC00152 promoter. The post-transcriptional regulatory prediction site POSTAR2 [47] was used to predict the potential RNA binding protein that interacted with LINC00152.

### Cell culture

A549, PC9, Calu3, H1975, H1299 and Human bronchial epithelial cells (HBE) were cultured in RPMI1640 (Gibco, Life Technology, Carlsbad, CA, USA). Human embryonic kidney cell line HEK293 and HEK293T cells were grown in DMEM medium (Gibco, Life Technology, Carlsbad, CA, USA). Both RPMI-1640 and DMEM medium were supplemented with 10% FBS and 1% penicillin/streptomycin and all cell lines were grown at 37 °C and 5% CO_2_. The cells were obtained from the cell bank of Cancer Research Institute of Central South University (Hunan, China) [11, 40].

For TGF-β treatment in cells, the cells were seeded in 6-well plates and cultured in medium containing 5% FBS. After 12 h, the medium was replaced with FBS-free medium to continue starvation culture for an additional 12 h before starting the treatment. TGF-β powder (Sino Biological, Beijing, China) dissolve in sterile water to prepare a stock solution of 0.1 mg/ml. Then, according to the experimental design, TGF-β solution was added into cells to make the final concentration. Cells were harvested for RNA extraction at different time points.

### Clinical specimens

The LUAD tissues with corresponding matched paracancerous tissue were obtained from surgical operation patients at the Second Xiangya Hospital, Central South University (Changsha, China). Written informed consent was provided by each enrolled subject. All samples were immediately snap-frozen in liquid nitrogen after surgical resection and then stored at −80 °C until RNA extraction. All specimens were confirmed by histopathological examination. The Human Research Ethics Committee of Xiangya Second Hospital granted the ethics approval for this study. All our methods and experimental protocols were according to the guidelines approved by the Human Research Ethics Committee of Xiangya Second Hospital. Clinic information was collected from patient medical records and reported in Table S2.

### Plasmid construction and cell transfection

To construct a plasmid expressing LINC00152, transcript variant 1 (NCBI:NR_024204.2) was synthesized and subcloned into the pcDNA3.1 vector (Sangon, Shanghai, China). In order to construct a luciferase expression vector containing the promoter region of LINC00152, we obtained the sequence from 2000 bp to 200 bp before the start site of the transcription of LINC00152 from NCBI. We designed primers to amplify the corresponding DNA fragments, and connected them to the PGL3-enhancer vector (PGL3-LINC00152). The SMAD3 plasmid was donated by Professor Ma Jian. The pcDNA3.1-HuR-Flag plasmid was purchased from HANBIO (Shanghai, China). The plasmids were transfected into cells using Lipofectamine3000 Reagent (Invitrogen^**TM**^, USA). The small interfering RNA used in the article was purchased from RiboBio (Guangzhou, China), and then transfect into cells with Hipfect transfection reagent (QIAGEN, Beijing, China). All primers used for plasmid construction and all siRNAs sequences were listed in Table S3.

### RNA isolation and RT-qPCR

Total RNA was extracted with RNeasy Mini kit (Foregene Biotec, Chengdu, China) according to the manufacturer’s protocol. The RNA quantity and quality was evaluated by Nano Drop ND-2000 spectrophotometer. 2 μg total RNA was transcribed into cDNA for LINC00152 expression analysis with the RevertAid First Strand cDNA Synthesis Kit (Thermo Scientific™, USA). RT-qPC*R* was performed with 2x SYBR Green qPCR Master Mix (Bimake, China) in Bio-Rad CFX Connect™ Real-Time PCR Detection System (Bio-Rad, USA). GAPDH was used as a control for RT-qPCR and the 2-ΔΔCt method was used to analysis the relative quantification of gene expression levels. Primers for RT-qPCR analysis were synthesized by Tsingke (Beijing, China). RT-qPCR primers used in this study and sequence of LINC00152 and HuR siRNA were listed in Table S3.

### CCK8 assay

Cells were transfected with si-LNC00152 or pcDNA3.1-LINC00152 for 48 h, and then seeded in a 96-well plate at a density of 800 cells/well. Changes in cell viability were determined by adding 20 μl of CCK8 solution (5 mg/ml) to each well at 0, 1, 2, 3, 4 and 5-day time points. The plate was incubated at 37 °C for an additional 2 h. The optical density of each well was determined with a scanning multi-well spectrophotometer at a wavelength of 450 nm. The experiences were repeated three times and six parallel samples were measured each time.

### Colony formation assay

Cells were trypsinized into a single-cell suspension after transfection with siRNA oligos targeting LNC00152 or pcDNA3.1-LINC00152 for 48 h. About 800 cells were plated in each well of the 6-well plate and maintained for 2 weeks to form colony. After fixation with 1% neutral formaldehyde, 0.1% crystal violet was added for staining, and the colonies with over 50 cells were counted after scanning under the scanner.

### Wound healing assay

After 48 h of transfection of si-LINC00152 or pcDNA3.1- LINC00152 in a 6-well plate, a wound was created using a 10 μl pipette tip followed by washing with D-Hanks to remove detached cells. Cells were then cultured in medium with 2% serum. Images were captured 0, 24 h hours after wounding using a microscope (Nikon). Image-Pro Plus 6.0 was used to analyze the picture and get the corresponding scratch distance (at least three randomly selected fields were imaged).

### Transwell migration assay

Corning costar transwell 24-well plates (8 μm pores; Corning, NY, USA) were coated with matrigel (Biolead, Beijing, China) and incubated at 37 °C for 30 min until the matrigel solidified. Pre-warmed medium with 10% serum was added to the lower chambers. Then cell suspension (2 × 10^4^ cells/well) was seeded to upper chamber in a 1640 medium. After incubation 48 h, the cells on the bottom surface of the membrane were fixed with cold methanol for 15 min and stained with crystal violet for 10 min.

### Western blot assay

The proteins were extracted using Radio Immunoprecipitation Assay Buffer (Beyotime Biotechnology, Haimen, China) with protease inhibitor (Selleck, USA). BCATM protein analysis kit (Thermo Fisher Scientific) was used to quantify the proteins. 50 μg protein was separate using a 10% sodium dodecyl sulfate-polyacrylamide gel electrophoresis and transferred to the PVDF membrane (Millipore). Membranes were blocked with 5% nonfat milk in 1 × TBST (TBST; 25 mM Tris pH 7.5, 150 mM NaCl, 0.1% Tween 20) for 1 h at room temperature, and then incubated with the diluted primary specific antibodies overnight at 4 °C. After washing with 1 × TBST, the membrane was incubated with the horseradish peroxidase-labeled secondary antibody at 37 °C for 1 h. The signal was visualized using an ECL detection reagent (Millipore, America) and quantified by densitometry (BioRad ChemiDoc XRS system). Information of antibodies used in this study is provided in Table S4.

### Dual-luciferase reporter assay

All luciferase reporter constructs were generated from the PGL3-basic vector (Promega, Madison, WI, USA). The primers used are shown in Table S3. To evaluate that the LINC00152 is directly targeted by SMAD3, cells were transfected with SMAD3 and PGL3-LINC00152 plasmid. Firefly and Renilla luciferase activity was examined by the Dual-Luciferase^®^ Reporter Assay System following the manufacturer’s instructions (Promega) after 48 h, and Renilla activity was used to normalize firefly activity.

### Chromatin immunoprecipitation

Chromatin immunoprecipitation (ChIP) assay was described previously [11]. Briefly, after 24 h of SMAD3 plasmid transfection, cells were cross-linked with 1% formaldehyde for 10 min at room temperature, and the fixation was stopped with 0.125 M glycine, the cell lysis buffer was then added and the samples were sonicated to generate 200–1000 bp fragments. The sonicated chromatin was immunoprecipitated with anti-SMAD3 antibody or control IgG (Santa Cruz Blotechnology, USA) at 4 °C overnight. Finally, DNA fragments were amplified with specially primers that recognize SMAD3 binding site with LINC00152 promoter (Table S3).

### RNA stability analysis

Actinomyicn D (Sigma, Missouri, USA) treatment were used to detect the stability of RNA. We dissolved the actinomycin D powder in DMSO. After TGF-β (10 ng/mL) induced for 2 h, A549 cells were treated with actinomycin D (10 μg/mL) for 0 h, 1 h, 2 h, 3 h, 4 h, 5 h, 6 h, 7 h, 8 h, 9 h or 10 h followed by the extraction of RNA with the Trizol reagent. RT-qPCR were performed using the 2^−ΔΔCt^ method and mRNA levels were normalized to the 0 h time point.

### RNA immunoprecipitation (RIP) assay

To verify the interaction between HuR and mRNA, we transfected the pcDNA3.1-HuR-Flag plasmid into A549 cells, then performed a RIP experiment using FLAG affinity gelbeads (A2220, Simga, USA) following the manufacturer’s protocol. Then RT-qPCR analysis was used to detect the enrichment of LINC00152, ZEB1, SNAI1 and SNAI2 on FLAG-beads. Normal mouse IgG (Millipore, USA) was used as a negative control.

### Biotinylated RNA pull-down assay

LINC00152 and LINC00152-antisense (negative control) cut by enzyme were transcribed with T7 RNA polymerase in the presence of the Biotin RNA Labeling Mix (Roche, Mannheim, Germany) in vitro. Add 2 μL of DNase I (Takara, Japan) to the obtained biotin-labeled RNA to remove the DNA and purify it with the RNeasy Mini Kit (Qiagen). Take 3 μg Biotin-RNA and Dynabeads™ M-270 Streptavidin (Invitrogen TM) for cross-linking 30 min, then incubate the cross-linked beads with cell lysates (Concentration ≥ 2 mg/mL) for 2 h at 25 °C. Finally, immunocomplexes were analyzed by western blotting.

### Knockout of LINC00152 by CRISPR/Cas9

We used a dual gRNA approach to knock out LINC00152 by CRISPR/Cas9 system. To facilitate the selection of positive clones, sgRNAs were designed in two donor vectors (lentiCRISPR-v2 and pLKO5.sgRNA.EFS.tRFP580) that were cotransfected with pMD.2 G and psPAX2 into the HEK293T cells in a 6-mm dish using Neofect™ DNA transfection reagent (Neofect (Beijing) biotech, China). 48 h later after transfection, a supernatant from lentivirus-producing cells filtered through 0.45 μm filters was added to six-well plates with A549 cells. One week later, the transfected cells were subject to puromycin selection; and surviving cells were sorted by FACS based on RFP580 signal into 96-well plates and then expanded. Initial identification of knockout (KO) clones was carried out by genomic PCR, followed by RT-qPCR. Oligonucleotide sequences of sgRNAs and primers used to choose CRISPR-LINC00152 A549 cells are shown in Table S3.

### Animal experiments

We purchased 4-week-old female BALB/c nude mice from the Hunan SJA Laboratory Animal Company (Hunan, China) and maintained under SPF conditions. Animal experiments were approved by the Institutional Animal Care and Use Committee of Hunan Cancer Hospital (Changsha, China). For lung metastasis experiments, nude mice were randomly divided into two groups (*n* = 3 per group). LINC00152 KO or negative control A549 cells were infected with a virus containing pLenti6 V5 D-TOPO Luciferase EGFP (CMV/Luciferase17-EGFP&BSD) vector and were injected into the tail veins of the nude mice (4 × 10^6^ cells per mice). At 8 weeks after injection, optical in vivo imaging of cancer metastasis was monitored with in vivo luminescence imaging system (IVIS). Nude mice were sacrificed by cervical dislocation. Lung tissue was removed and the number of nodules on the surface of the lung was recorded to assess tumor metastasis. For subcutaneous tumorigenesis experiments, nude mice were randomly divided into two groups (*n* = 8 per group). Each nude mouse was subcutaneously injected with the LINC00152 KO and negative control A549 cells (5 × 10^6^). Tumor growth was monitored every 3 days. Tumor size was assessed by measuring the largest perpendicular diameters, and tumor volume was calculated as follows: V = 1/2 × (length) × (width) × (width). 36 days after subcutaneous inoculation, mice were euthanized by cervical dislocation, and the tumor tissue was excised. The formed tumor masses were removed and weighed. All photographed tumors were isolated from single experiments at a similar time points. Part of the subcutaneous tumors were detected the expression of LINC00152 by RT-qPCR, and EMT associated genes (ZEB1, Slug, ZO-1, Vimentin) by Western blot.

### Statistical analysis

All statistical analysis were performed using SPSS 24.0 software (SPSS, IBM, Armonk, NY, USA) and GraphPad Prism V8.0 (GraphPad Software, CA, USA). Significant differences between any two groups of data were made using Student’s *t*-test. One-way analysis of variance was used when assessing significant differences between multiple sets of data. The cumulative overall survival (OS) was calculated using the Kaplan–Meier method, and the log-rank test was used to analyze differences in the survival times. All data are presented as mean ± Standard Deviation (SD). Differences were considered significant at *p < 0.05; **p < 0.01; ***p < 0.001; ****p < 0.0001.

## Supplementary information

Table S1

Table S2

Table S3

Table S4

Supplementary Figure Legends

Fig. S1

Fig. S2

Fig. S3

Fig. S4

Checklist

Original Data File

## Data Availability

Data used or analyzed during the current study are available from the corresponding author on reasonable request.

## References

[1] Norouzi M, Hardy P (2021). Clinical applications of nanomedicines in lung cancer treatment. Acta Biomater.

[2] Oudkerk M, Liu S, Heuvelmans MA, Walter JE, Field JK (2021). Lung cancer LDCT screening and mortality reduction - evidence, pitfalls and future perspectives. Nat Rev Clin Oncol.

[3] Sung H, Ferlay J, Siegel RL, Laversanne M, Soerjomataram I, Jemal A (2021). Global Cancer Statistics 2020: GLOBOCAN estimates of incidence and mortality worldwide for 36 cancers in 185 countries. CA Cancer J Clin.

[4] Denisenko TV, Budkevich IN, Zhivotovsky B (2018). Cell death-based treatment of lung adenocarcinoma. Cell Death Dis.

[5] Fang L, Li Z, Chen YJ, Xiao GM (2017). Cyramza induces apoptosis of HCC4006 cell by affecting the level of Bcl-w. Eur Rev Med Pharm Sci.

[6] Man J, Zhang X, Dong H, Li S, Yu X, Meng L (2019). Screening and identification of key biomarkers in lung squamous cell carcinoma by bioinformatics analysis. Oncol Lett.

[7] Sun R, Wang R, Chang S, Li K, Sun R, Wang M (2019). Long non-coding RNA in drug resistance of non-small cell lung cancer: a mini review. Front Pharm.

[8] Saito A, Horie M, Nagase T (2018). TGF- Signaling in Lung Health and Disease. Int J Mol Sci.

[9] Toonkel RL, Borczuk AC, Powell CA (2010). Tgf-beta signaling pathway in lung adenocarcinoma invasion. J Thorac Oncol.

[10] Lai X-N, Li J, Tang L-B, Chen W-T, Zhang L, Xiong L-X (2020). MiRNAs and LncRNAs: dual roles in TGF-á signaling-regulated metastasis in lung cancer. Int J Mol Sci.

[11] Jiang L, Wang R, Fang L, Ge X, Chen L, Zhou M (2019). HCP5 is a SMAD3-responsive long non-coding RNA that promotes lung adenocarcinoma metastasis via miR-203/SNAI axis. Theranostics.

[12] Yuan JH, Yang F, Wang F, Ma JZ, Guo YJ, Tao QF (2014). A long noncoding RNA activated by TGF-beta promotes the invasion-metastasis cascade in hepatocellular carcinoma. Cancer Cell.

[13] Xu L, Liu W, Li T, Hu Y, Wang Y, Huang L (2021). Long non-coding RNA SMASR inhibits the EMT by negatively regulating TGF-β/Smad signaling pathway in lung cancer. Oncogene.

[14] Sarkar A, Rahaman A, Biswas I, Mukherjee G, Chatterjee S, Bhattacharjee S (2020). TGFβ mediated LINC00273 upregulation sponges mir200a-3p and promotes invasion and metastasis by activating ZEB1. J Cell Physiol.

[15] Liu J, Zhang C, Jia X, Wang W, Yin H (2020). Comparative analysis of RNA-binding proteomes under Arabidopsis thaliana-Pst DC3000-PAMP interaction by orthogonal organic phase separation. Int J Biol Macromol.

[16] Modic M, Grosch M, Rot G, Schirge S, Lepko T, Yamazaki T (2019). Cross-regulation between TDP-43 and Paraspeckles promotes Pluripotency-differentiation transition. Mol Cell..

[17] Kafasla P, Skliris A, Kontoyiannis DL (2014). Post-transcriptional coordination of immunological responses by RNA-binding proteins. Nat Immunol.

[18] Tripathi V, Sixt KM, Gao S, Xu X, Huang J, Weigert R (2016). Direct regulation of alternative splicing by SMAD3 through PCBP1 is essential to the tumor-promoting role of TGF-β. Mol Cell.

[19] Zhang J, Li S, Zhang L, Xu J, Song M, Shao T (2020). RBP EIF2S2 promotes tumorigenesis and progression by regulating MYC-mediated inhibition via FHIT-related enhancers. Mol Ther: J Am Soc Gene Ther.

[20] Hu Y-P, Jin Y-P, Wu X-S, Yang Y, Li Y-S, Li H-F (2019). LncRNA-HGBC stabilized by HuR promotes gallbladder cancer progression by regulating miR-502-3p/SET/AKT axis. Mol Cancer.

[21] Spizzo R, Almeida MI, Colombatti A, Calin GA (2012). Long non-coding RNAs and cancer: a new frontier of translational research?. Oncogene.

[22] Jiang G, Yu H, Li Z, Zhang F (2021). lncRNA cytoskeleton regulator reduces non-small cell lung cancer radiosensitivity by downregulating miRNA-206 and activating prothymosin α. Int J Oncol.

[23] Chen Q-N, Chen X, Chen Z-Y, Nie F-Q, Wei C-C, Ma H-W (2017). Long intergenic non-coding RNA 00152 promotes lung adenocarcinoma proliferation via interacting with EZH2 and repressing IL24 expression. Mol Cancer.

[24] Wang X, Yu H, Sun W, Kong J, Zhang L, Tang J (2018). The long non-coding RNA CYTOR drives colorectal cancer progression by interacting with NCL and Sam68. Mol Cancer.

[25] Yue B, Liu C, Sun H, Liu M, Song C, Cui R (2018). A positive Feed-Forward Loop between LncRNA-CYTOR and Wnt/β-Catenin signaling promotes metastasis of Colon Cancer. Mol Ther.

[26] Liu D, Gao M, Wu K, Zhu D, Yang Y, Zhao S (2019). LINC00152 facilitates tumorigenesis in esophageal squamous cell carcinoma via miR-153-3p/FYN axis. Biomed Pharmacother.

[27] Chen S, Yang M, Wang C, Ouyang Y, Chen X, Bai J (2021). Forkhead box D1 promotes EMT and chemoresistance by upregulating lncRNA CYTOR in oral squamous cell carcinoma. Cancer Lett.

[28] Ou C, Sun Z, He X, Li X, Fan S, Zheng X (2020). Targeting YAP1/LINC00152/FSCN1 Signaling Axis Prevents the Progression of Colorectal Cancer. Adv Sci (Weinh).

[29] Zhao Y, Liu Y, Lin L, Huang Q, He W, Zhang S (2018). The lncRNA MACC1-AS1 promotes gastric cancer cell metabolic plasticity via AMPK/Lin28 mediated mRNA stability of MACC1. Mol Cancer.

[30] Chae KS, Kang MJ, Lee JH, Ryu BK, Lee MG, Her NG (2011). Opposite functions of HIF-α isoforms in VEGF induction by TGF-β1 under non-hypoxic conditions. Oncogene.

[31] Dong R, Chen P, Polireddy K, Wu X, Wang T, Ramesh R (2020). An RNA-Binding Protein, Hu-antigen R, in Pancreatic Cancer Epithelial to Mesenchymal Transition, Metastasis, and Cancer Stem Cells. Mol Cancer Ther.

[32] Chen S, Shen X (2020). Long noncoding RNAs: functions and mechanisms in colon cancer. Mol Cancer.

[33] Tang R, Zhang G, Wang Y-C, Mei X, Chen S-Y (2017). The long non-coding RNA GAS5 regulates transforming growth factor β (TGF-β)-induced smooth muscle cell differentiation via RNA Smad-binding elements. J Biol Chem.

[34] Tang R, Zhang G, Wang YC, Mei X, Chen SY (2017). The long non-coding RNA GAS5 regulates transforming growth factor beta (TGF-beta)-induced smooth muscle cell differentiation via RNA Smad-binding elements. J Biol Chem.

[35] Tripathi V, Shin J-H, Stuelten CH, Zhang YE (2019). TGF-β-induced alternative splicing of TAK1 promotes EMT and drug resistance. Oncogene.

[36] Su Y, Feng W, Shi J, Chen L, Huang J, Lin T (2020). circRIP2 accelerates bladder cancer progression via miR-1305/Tgf-β2/smad3 pathway. Mol Cancer.

[37] Shen Q, Zhang Q, Shi Y, Shi Q, Jiang Y, Gu Y (2018). Tet2 promotes pathogen infection-induced myelopoiesis through mRNA oxidation. Nature.

[38] Cox DC, Guan X, Xia Z, Cooper TA (2020). Increased nuclear but not cytoplasmic activities of CELF1 protein leads to muscle wasting. Hum Mol Genet.

[39] Ge J, Chang N, Zhao Z, Tian L, Duan X, Yang L (2016). Essential roles of RNA-binding protein HuR in activation of Hepatic Stellate Cells induced by transforming growth factor-beta1. Sci Rep.

[40] Ge X, Li G-Y, Jiang L, Jia L, Zhang Z, Li X (2019). Long noncoding RNA CAR10 promotes lung adenocarcinoma metastasis via miR-203/30/SNAI axis. Oncogene.

[41] Chen QN, Chen X, Chen ZY, Nie FQ, Wei CC, Ma HW (2017). Long intergenic non-coding RNA 00152 promotes lung adenocarcinoma proliferation via interacting with EZH2 and repressing IL24 expression. Mol Cancer.

[42] Sartor MA, Mahavisno V, Keshamouni VG, Cavalcoli J, Wright Z, Karnovsky A (2010). ConceptGen: a gene set enrichment and gene set relation mapping tool. Bioinformatics.

[43] Van Grembergen O, Bizet M, de Bony EJ, Calonne E, Putmans P, Brohee S (2016). Portraying breast cancers with long noncoding RNAs. Sci Adv.

[44] Yamauchi M, Yamaguchi R, Nakata A, Kohno T, Nagasaki M, Shimamura T (2012). Epidermal growth factor receptor tyrosine kinase defines critical prognostic genes of stage I lung adenocarcinoma. PLoS One.

[45] Bryne JC, Valen E, Tang MH, Marstrand T, Winther O, da Piedade I (2008). JASPAR, the open access database of transcription factor-binding profiles: new content and tools in the 2008 update. Nucleic Acids Res.

[46] Grant CE, Bailey TL, Noble WS (2011). FIMO: scanning for occurrences of a given motif. Bioinformatics.

[47] Zhu Y, Xu G, Yang YT, Xu Z, Chen X, Shi B (2019). POSTAR2: deciphering the post-transcriptional regulatory logics. Nucleic Acids Res.

