# Supplementary material for: LINC00152 induced by TGF-β promotes metastasis via HuR in lung adenocarcinoma
Source: Cell Death Dis. 2022 Sep 7;13(9):772. doi: 10.1038/s41419-022-05164-2 (PMC9452677; doi:10.1038/s41419-022-05164-2)
Supplement: Supplementary file 5 — Supplementary Figure Legends [file 41419_2022_5164_MOESM5_ESM.docx]

**Supplementary Figure legends**

**Fig. S1. Expression and survival analysis of 12 lncRNAs in human LUAD tissue and normal in GSE31210.**

**Fig. S2. Expression and survival analysis of 12 lncRNAs in human LUAD tissue and normal in GSE30219.**

**Fig. S3. Relationship between HuR and LINC00152.**

**(A)** The proteins binding with LINC00152 predicted by the PAR-CLIP dataset in POSTAR2. **(B)** The overall survival rates of LUAD patients in TCGA according to the expression level of HuR. **(C)** Expression of HuR in human LUAD samples and paired normal tissues in GEPIA website. **(D)** After transfection with siLINC00152 or LINC00152 overexpression plasmids in A549 cells, RT-qPCR was used to detect the expression level of HuR mRNA. Data have been presented as the mean ± SD of three independent experiments (**, *p* < 0.01; ***, *p* < 0.001; ****, *p* < 0.00001; Student’s t-test).

**Fig. S4. Knockout of LINC00152 exon1 by CRISPR/Cas9 system.**

**(A)** Two gRNAs were designed to delete the entire exon 1 of LINC00152. **(B)** Schematic diagram of CRISPR LINC00152 vector construction. **(C)** PCR analysis of the targeted locus showing the band corresponding to the genomic deletion in cell colonies, and a knockout-positive colony of A549 cells was chosen for further research. **(D)** RT-qPCR analysis of LINC00152 expression in A549 cells treated with LINC00152 KO#2. **(E)** Suppression of cell invasion in LINC00152 KO cells, as determined by transwell. **(F)** Detection of the expression of EMT markers in LINC00152 KO group and control group at the cellular level. **(G)** Tumor-bearing mice of LINC00152 KO group and control group were photographed. Data have been presented as the mean ± SD of three independent experiments (**, *p* < 0.01; ***, *p* < 0.001; ****, *p* < 0.00001. Student’s t-test).
