# Supplementary figures and images for: LINC00152 induced by TGF-β promotes metastasis via HuR in lung adenocarcinoma
Source: Cell Death Dis. 2022 Sep 7;13(9):772. doi: 10.1038/s41419-022-05164-2 (PMC9452677; doi:10.1038/s41419-022-05164-2)

**Fig. 3C**

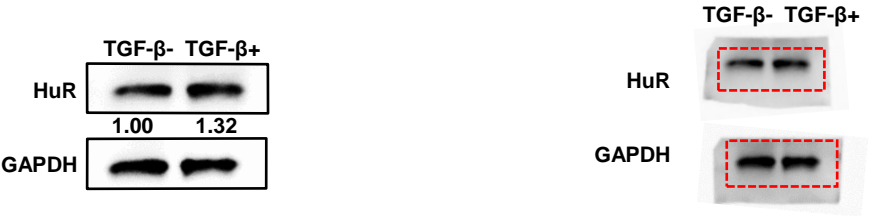

**Fig. 3D**

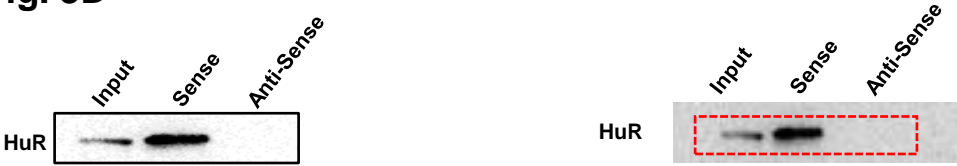

**Fig. 6D**

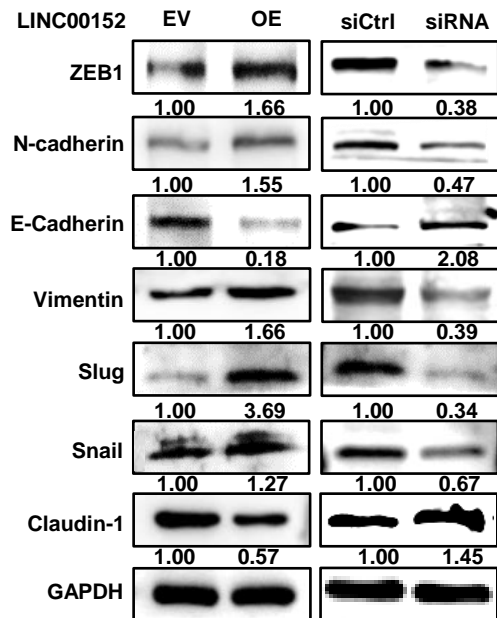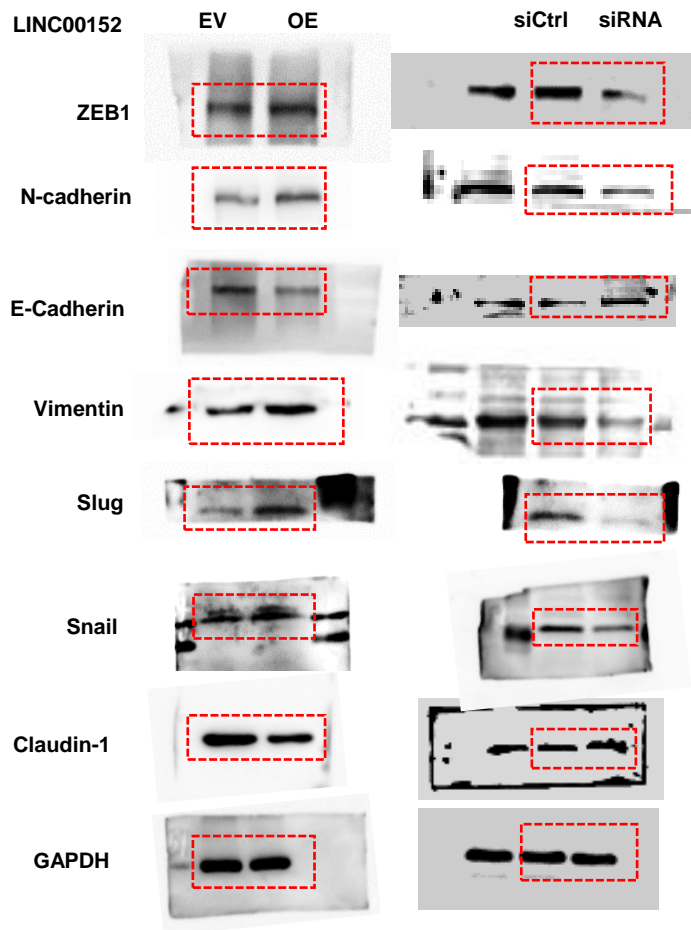

**Fig. 7B**

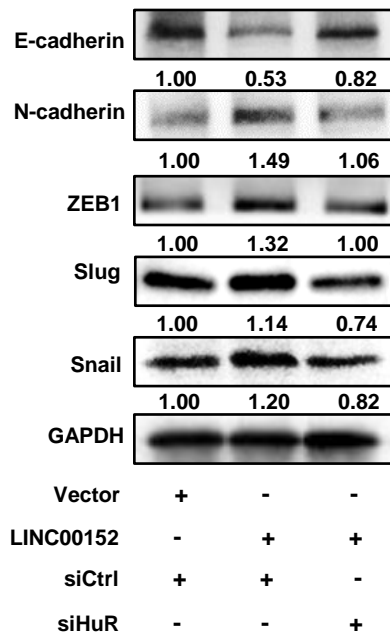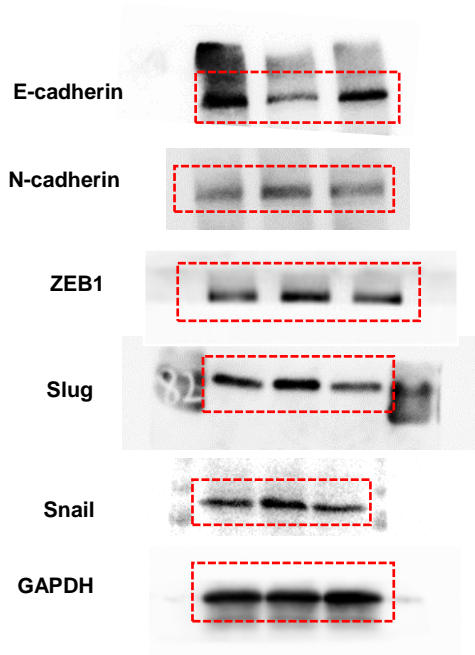

**Fig. 8G**

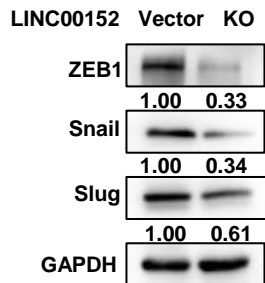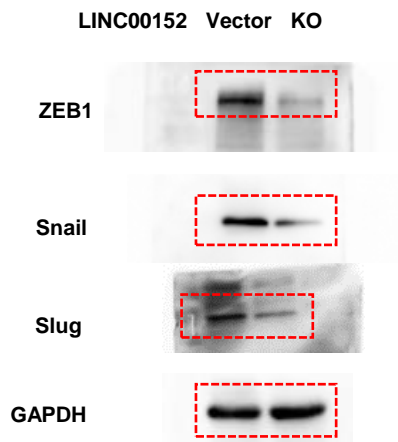

**Fig. S3E**

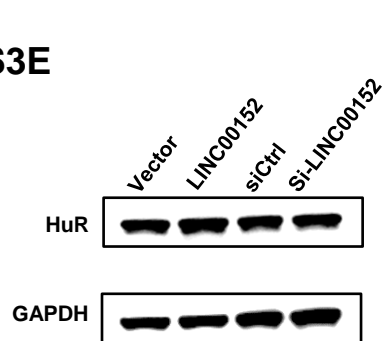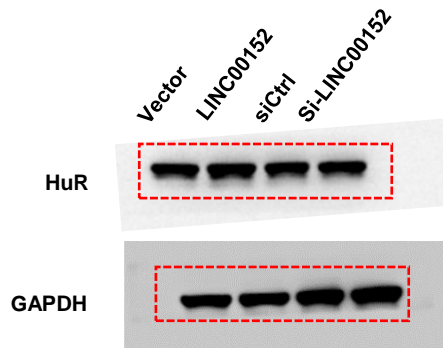

**Fig. S4F**

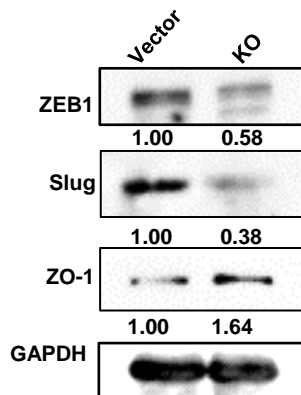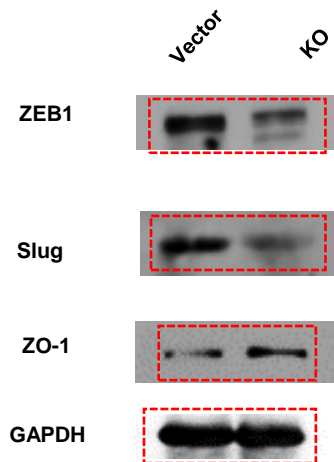

Supplement: Supplementary file 11 — Original Data File [file 41419_2022_5164_MOESM11_ESM.pdf]
